# Successful preterm delivery in a patient with bicornuate uterus and right renal agenesis: Diagnostic and clinical perspectives

**DOI:** 10.1016/j.radcr.2025.09.066

**Published:** 2025-10-21

**Authors:** Soheil Mirzaei, Zahra Motaghed

**Affiliations:** a Department of Anatomy, Faculty of Medical Sciences, Tarbiat Modares University, Tehran, Iran; b Department of Radiology, Shahid Sattari Hospital, Shahid Beheshti University of Medical Sciences, Tehran, Iran

**Keywords:** Müllerian anomalies, Bicornuate uterus, Renal agenesis

## Abstract

Müllerian duct anomalies can occur during embryogenesis as complex developmental defects of the uterus and other female reproductive organs. This article reports a rare case of bicornuate uterus and right renal agenesis. A 39-year-old patient with a history of successful pregnancy presented to the hospital emergency department with symptoms of abdominal pain and nausea. Imaging Finding revealed a bicornuate uterus and right renal agenesis. The patient was discharged after supportive treatment with improved symptoms. This case describes congenital anomalies of a bicornuate uterus and right renal agenesis along with imaging findings and clinical correlations. Uterine anomalies can be associated with obstetric complications and infertility. Accurate diagnosis and management of uterine anomalies are of great importance. The use of advanced imaging techniques and proper education about Müllerian anomalies can help improve patient care and prevent complications.

## Introduction

**Fig. 1:**
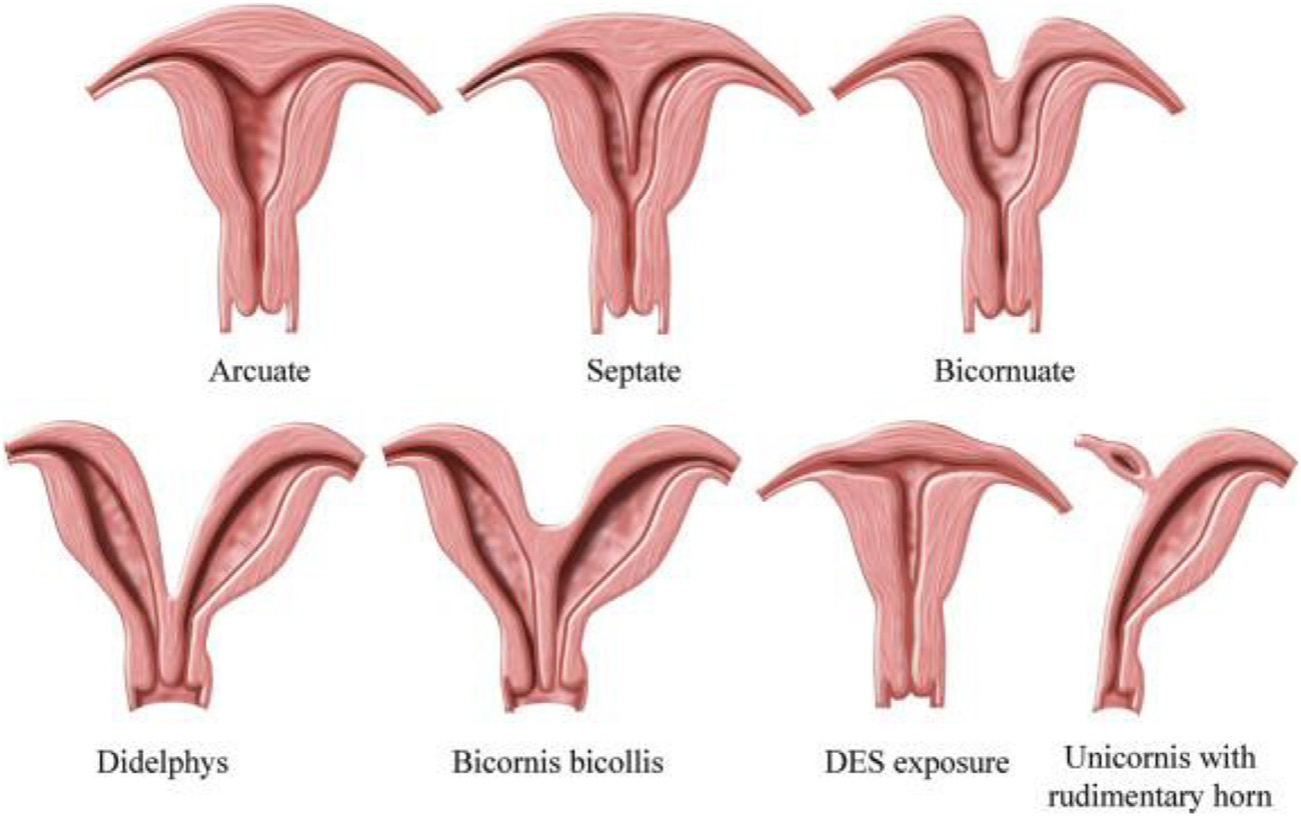
Most common uterine anomalies. DES: diethylstilbestrol [7].

Müllerian duct anomalies can manifest during embryogenesis as abnormalities or unsuccessful developments in the uterus, cervix, and proximal vagina. These anomalies include hypoplasia or agenesis of the uterus, unicornuate uterus, bicornuate uterus, didelphys uterus, septate uterus, arcuate uterus, and diethylstilbestrol (DES) uterus (Fig. 1). They may be asymptomatic or present with infertility issues and obstetric complications [1]. The prevalence of uterine anomalies in the general population is estimated to be between 1% to 4%, with the bicornuate uterus accounting for nearly half of these anomalies. This congenital defect is caused by the incomplete or absent development of the female reproductive system during embryogenesis [2]. The normal development of the female reproductive system involves a series of complex processes characterized by differentiation, migration, fusion, and canalization of the Müllerian system [3]. Müllerian anomalies represent a complex array of developmental defects, occurring in approximately 5% of the general population. This percentage increases to 8% in individuals with infertility and to 13.3% in those with a history of pregnancy loss. The highest prevalence of these defects, 24.5%, is seen in individuals with both histories [4]. A bicornuate uterus is one of the Müllerian anomalies where the uterine fundus is indented, and the cervix and vagina are usually normal. This defect arises due to incomplete fusion of the Müllerian ducts at the level of the uterine fundus, resulting in two separate but communicating endometrial cavities and a single cervix [3]. Imaging plays a crucial role in the accurate diagnosis of Müllerian duct anomalies and the identification of associated urinary tract anomalies [1].

## Case presentation

**Fig. 2:**
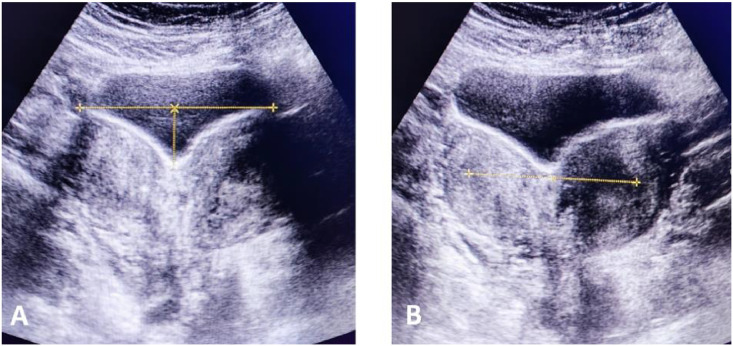
Coronal Transabdominal sonography (TAS) view of a bicornuate uterus and the deep intervening cleft between the 2 horns—(A) fundal cleft depth of 1 cm or more between the horns, (B) intercornual distance greater than 4 cm.

**Fig. 3:**
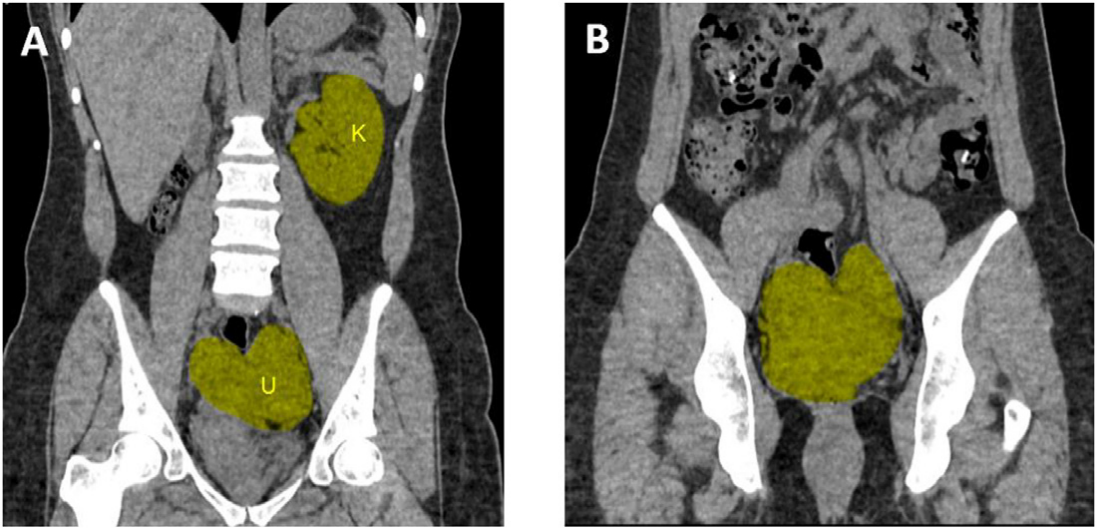
Coronal CT scan of the abdomen and pelvis showing (A) Müllerian anomaly as a bicornuate uterus (U) and absence of the right kidney—highlighted areas, (B) bicornuate uterus (highlighted area).

A 39-year-old woman with a history of successful pregnancy and delivery at 29 weeks (preterm delivery) presented to the hospital emergency department with complaints of abdominal pain and nausea. Physical examination of the abdomen revealed normal findings, and initial laboratory tests were also normal. Following these examinations, CT scan and ultrasound were performed, revealing a bicornuate uterus and right renal agenesis, indicative of congenital anomalies. Imaging findings showed an echogenic debris-containing bladder, suggesting the need for further evaluation for cystitis with a U/A and U/C check. Additionally, the uterus measured 84*32 mm with a heterogeneous echo pattern and contained 2 endometrial cavities and a myometrial indentation, consistent with a bicornuate uterus. The endometrial thickness was 6 mm, which is within normal limits, and the ovaries exhibited polycystic ovary (PCO) morphology, characterized by enlarged size, central echogenic stroma, and multiple small peripheral follicles, indicative of polycystic ovary morphology (PCOM) (Fig. 2, Fig. 3). According to information recently obtained from the patient, the expected due date was in November 2021, but the delivery occurred prematurely in September 2021. The newborn was admitted to the neonatal intensive care unit (NICU) following birth, and the delivery was ultimately successful. At the time of her presentation to the emergency department, approximately four years had passed since the delivery. However, the patient was clinically stable, and her current visit was due to new-onset abdominal complaints. Regarding her pregnancy history, the patient provided limited information and mentioned that she had previously been told her chances of conceiving were very low. Nevertheless, she did not provide any documentation or reports from prenatal ultrasounds. According to information recently obtained from the patient, the expected due date was in November 2021, but the delivery occurred prematurely in September 2021. The newborn was admitted to the neonatal intensive care unit (NICU) following birth, and the delivery was ultimately successful. At the time of her presentation to the emergency department, approximately four years had passed since the delivery. However, the patient was clinically stable, and her current visit was due to new-onset abdominal complaints. Regarding her pregnancy history, the patient provided limited information and mentioned that she had previously been told her chances of conceiving were very low. Nevertheless, she did not provide any documentation or reports from prenatal ultrasounds. According to information recently obtained from the patient, the expected due date was in November 2021, but the delivery occurred prematurely in September 2021. The newborn was admitted to the neonatal intensive care unit (NICU) following birth, and the delivery was ultimately successful. At the time of her presentation to the emergency department, approximately four years had passed since the delivery. However, the patient was clinically stable, and her current visit was due to new-onset abdominal complaints. Regarding her pregnancy history, the patient provided limited information and mentioned that she had previously been told her chances of conceiving were very low. Nevertheless, she did not provide any documentation or reports from prenatal ultrasounds. After undergoing necessary diagnostic evaluations and supportive treatment, the patient was discharged with improved symptoms. This case report explores and describes the congenital anomalies of a bicornuate uterus and right renal agenesis, along with ultrasound and CT scan findings, and their clinical correlations.

## Clinical discussion

A bicornuate uterus is a rare uterine anomaly that occurs due to the incomplete fusion of the Müllerian ducts, leading to the formation of a heart-shaped uterus instead of the typical pear shape [5]. This condition can be associated with issues such as first and second-trimester pregnancy loss, preterm birth, abnormal fetal positions, and low birth weight infants [6]. Patients with a bicornuate uterus are usually asymptomatic but have a higher risk of obstetric complications such as miscarriage, preterm birth, intrauterine growth restriction, and malpresentation of the fetus [1]. Diagnosing pregnancy in a bicornuate uterus is rare, and this anomaly often remains asymptomatic and may go undetected without prenatal care [2].

In a bicornuate uterus, the intercornual distance is greater than 4 cm, and the fundal cleft depth is 1 cm or more. On ultrasound, the endometrial cavities are widely separated, and a deep indentation is observed in the fundal contour. This anomaly is associated with cervical incompetence and poor fetal outcomes. A bicornuate uterus has a single cervix, whereas a didelphys uterus has 2 cervical canals and may have a vaginal septum. Differentiation between a didelphys uterus and a bicornuate uterus is based on the presence of 2 cervical canals and a vaginal septum. In contrast, the fundal contour in a septate uterus is convex, flat, or indented less than 1 cm between the 2 endometrial cavities. The septum may be partial or complete and can extend into the cervical canal. This septum may contain fibrous or myometrial tissue, and many women with a septate uterus experience recurrent miscarriages and preterm labor [7].

The gold standard for diagnosing a bicornuate uterus is 3-dimensional transvaginal ultrasound or MRI. Detailed evaluation of the uterine septum by experienced individuals using 3-dimensional ultrasound has high reproducibility in diagnosing uterine anomalies, especially septate and bicornuate uterus, for preoperative planning [3]. MRI continues to be recognized as the gold standard imaging technique for assessing Müllerian duct anomalies. Several studies have reported associations between unicornuate uterus and ovarian anomalies, such as the absence of an ovary or ovaries located outside their usual position [6]. Congenital uterine anomalies are significantly associated with recurrent pregnancy loss, low birth weight, preterm birth, abnormal fetal positions, cesarean delivery, and uterine rupture [3].

Unilateral Renal Agenesis (URA) (4 in 100, 000 births) may occur due to the following reasons: incomplete development of the mesonephric ducts or incomplete development of the ureteral buds, leading to the failure of ureteral buds to induce metanephric blastema [6]. It may also be associated with genital anomalies such as the absence of the vas deferens, unicornuate or bicornuate uterus, skeletal anomalies, cryptorchidism, and cardiovascular anomalies [6]. Renal anomalies may accompany Müllerian agenesis, with an occurrence rate of up to 40%, while in cases of obstructed hemivagina, ipsilateral renal agenesis or renal anomalies may occur in up to 95% of cases [8]. The combination of invasive mucinous adenocarcinoma of the ovary with unilateral renal agenesis and a bicornuate uterus is rare. In the early stages of the disease, ovarian mucinous carcinoma has a good prognosis, but in advanced stages, the prognosis is poorer [6]. URA is not only associated with other congenital anomalies of the kidney and urinary tract (CAKUT), but also with Müllerian system anomalies, such as Herlyn-Werner-Wunderlich Syndrome (HWWS) and OHVIRA Syndrome. This association is due to embryology, as the vagina is formed by the interaction between the Müllerian tubules and the mesonephric ducts. Anomalies of the Wolffian ducts lead to renal agenesis or hypoplasia and uterine anomalies [9]. Solid pseudopapillary neoplasm (SPN), a rare and low-grade malignant tumor of the pancreas, has a very good prognosis and is observed in young Asian and African-American women. This tumor may co-occur with urogenital anomalies, requiring focused investigations, and SPN can affect any part of the pancreas [5]. The ASRM 2021 (MAC2021) classification of Müllerian anomalies divides these anomalies into 9 different categories. Each anomaly can be described using specific anatomical terms [8].

❖ M€ullerian agenesis
❖ Cervical agenesis
❖ Unicornuate uterus
❖ Uterus didelphys
❖ Bicornuate uterus
❖ Septate uterus
❖ Longitudinal vaginal septum
❖ Transverse vaginal septum
❖ Complex anomalies

URA is typically identified as an incidental radiological diagnosis, where 1 in 3 young women with renal agenesis is associated with significant anomalies in the uterus, ovaries, or vagina [5]. One case report mentions a 32-year-old patient with a bicornuate uterus who achieved a viable pregnancy in one of the uterine horns and eventually delivered a healthy male infant via spontaneous vaginal delivery (SVD) without complications [10]. T. Sayer and colleagues reported 3 cases of genital anomalies associated with unilateral renal agenesis, where each case also included a single abnormal kidney, a rare occurrence [11]. Another case report depicted a rare combination of invasive mucinous ovarian carcinoma, unilateral renal agenesis, and a double uterus. This combination is generally considered a new triad, as the concurrence of these 3 conditions in 1 individual has not been fully investigated [12]. Early diagnosis of uterine and vaginal anomalies enables timely counseling for the patient and her family regarding the anomalies and treatment options to prevent menstrual complaints and infertility complications [9]. In cases of uterine anomalies, cesarean section is the preferred method of delivery [2]. Successful outcomes can be achieved in patients with a bicornuate uterus in post-term pregnancies, and if no other factors jeopardize fertility, the live birth rate gradually increases in women with a bicornuate uterus [3].

It is recommended that renal imaging be performed rigorously in the presence of Müllerian agenesis and unilateral obstructed vaginal and uterine anomalies. The set of presented anomalies is not meant to represent all known anomalies but includes most described anomalies. The new classification should encompass the diversity of many known anomalies and raise awareness of the potential to identify new variations in the future, enhancing overall patient care through education and accessibility [8].

## Conclusion

A bicornuate uterus is a rare anomaly that can be associated with various obstetric complications. Accurate diagnosis and evaluation using advanced imaging techniques can aid in the better management of these anomalies. Proper classification and education about Müllerian anomalies can improve patient care and prevent complications.

## Ethical approval

We obtained written permission from the patient to present this case report and to include images in the article, a written copy of which is available for submission to the journal, if necessary.

## Patient consent

Written informed consent was obtained from the patient for publication of this case report and accompanying images. A copy of the written consent is available for review by the Editor-in-Chief of this journal on request.
